# Supplementary material for: A loss-of-function variant in GFRAL associates with increased alcohol consumption in humans
Source: bioRxiv. 2026 Mar 9:2026.03.06.709997. Preprint. [Version 1] doi: 10.64898/2026.03.06.709997 (PMC13060954; doi:10.64898/2026.03.06.709997)
Supplement: Supplement 1 — Supplementary Figure 1: Topology information on GFRAL from UniProt Database. Uniprot topology information https://www.uniprot.org/uniprot/Q6UXV0. The rs527905870 results in frameshift of GFRAL protein structure at amino acid position 339 (p.Ile339AsnfsTer36) resulting in a GFRAL receptor possibly lacking the transmembrane and cytoplasmic parts of the receptor. Supplementary Figure 2: Scattershot showing genotype calling for GFRAL rs527905870 variant in UK Biobank http://mccarthy.well.ox.ac.uk/static/software/scattershot/ Genotype calling for the rs527905870 in white British individuals from UK Biobank (N = 409634) (Affx-52342012). There are 795 of the heterozygous genotype C/CA of rs527905870 among White British individuals. Supplementary Table 1: Population frequencies for GFRAL rs527905870 from gnomAD Population frequency for GFRAL rs527905870 in the UK Biobank (minor allele frequency (MAF) = 0.00096) is comparable to the gnomAD European population (MAF = 0.0014). https://gnomad.broadinstitute.org/variant/6-55264036-C-CA?dataset=gnomad_r2_1 Supplementary Table 2: Results from a Phenome-wide association study (PheWAS) of GFRAL rs527905870 in white British individuals from UK Biobank (p < 1×10^−4). Analyses including diseases with more than 100 cases. OR, odds ratio; SE, standard error. [file NIHPP2026.03.06.709997v1-supplement-1.pdf]

bioRxiv preprint doi: https://doi.org/10.64898/2026.03.06.709997; this version posted March 9, 2026. The copyright holder for this preprint (which was not certified by peer review) is the author/funder, who has granted bioRxiv a license to display the preprint in perpetuity. It is made available under aCC-BY 4.0 International license.

Supplemental Information

Supplementary Table 1. Population frequencies for *GFRAL* rs527905870 from gnomAD

**Population**	**Allele count**	**Allele number**	**Allele frequency**
African	7	24752	0.0002828
Ashkenazi Jewish	46	10328	0.004454
East Asian	0	19816	0.000
European (Finnish)	0	25068	0.000
**European (non-Finnish)**	**182**	**128426**	**0.001417**
Latino	33	35180	0.0009380
South Asian	5	30348	0.0001648
Other	10	7170	0.001395
Female	137	128510	0.001066
Male	146	152578	0.0009569
**Total**	**283**	**281088**	**0.001007**

Population frequency for *GFRAL* rs527905870 in UK Biobank (minor allele frequency (MAF) = 0.00096) is similar to gnomAD European population (MAF = 0.0014).

https://gnomad.broadinstitute.org/variant/6-55264036-C-CA?dataset=gnomad_r2_1

bioRxiv preprint doi: https://doi.org/10.64898/2026.03.06.709997; this version posted March 9, 2026. The copyright holder for this preprint (which was not certified by peer review) is the author/funder, who has granted bioRxiv a license to display the preprint in perpetuity. It is made available under aCC-BY 4.0 International license.

Supplementary Table 2: Results from a Phenome-wide association study (PheWAS) of *GFRAL* rs527905870 in white British individuals from UK Biobank.

**Phenotype name**	**Cases**	**Controls**	**OR/Beta**	**SE**	***p*-value**
Fracture thumb	424	336965	1.98	0.414	1.63e-06
Gastrointestinal bleeding	134	336965	2.47	0.587	2.52e-05
Ease of skin tanning (Never tan, only burn)	59404	330847	0.348	0.095	2.61e-04
Eye infection	358	336965	1.775	0.505	4.34e-04
Crumbed or deep-fried poultry intake (Diet, 24hr recall, Online follow-up)	9169	9167	-0.543	0.159	6.63e-04
Bile duct obstruction / Ascending cholangitis	1312	336965	1.180	0.357	9.60e-04

Analyses including diseases/traits with more than 100 cases. OR, odds ratio; SE, standard error. Only phenotypes with $p < 1x10^{-4}$ are presented.

bioRxiv preprint doi: https://doi.org/10.64898/2026.03.06.709997; this version posted March 9, 2026. The copyright holder for this preprint (which was not certified by peer review) is the author/funder, who has granted bioRxiv a license to display the preprint in perpetuity. It is made available under aCC-BY 4.0 International license.

Supplementary figures

Supplementary Figure 1. Topology information on GFRAL from UniProt Database.

Topology

Feature key	Position(s)	Description	Actions	Graphical view	Length
Topological domain i	19 – 351	Extracellular Sequence analysis	Add BLAST		333
Transmembrane i	352 – 371	Helical Sequence analysis	Add BLAST		20
Topological domain i	372 – 394	Cytoplasmic Sequence analysis	Add BLAST		23

Uniprot topology information https://www.uniprot.org/uniprot/Q6UXV0. The rs527905870 results in frameshift of GFRAL protein structure at amino acid position 339 (p.Ile339AsnfsTer36) resulting in a GFRAL receptor possibly lacking the transmembrane and cytoplasmic parts of the receptor.

Supplementary Figure 2. Scattershot showing genotype calling for *GFRAL* rs527905870 variant in UK Biobank

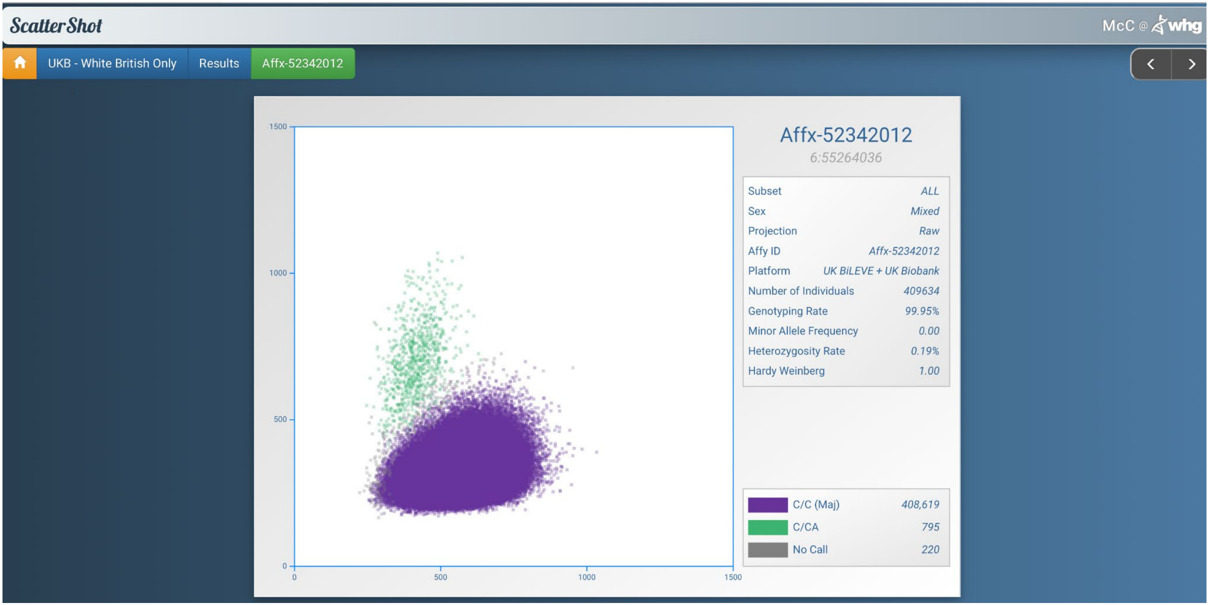

http://mccarthy.well.ox.ac.uk/static/software/scattershot/

Genotype calling for the rs527905870 in white British individuals from UK Biobank (N = 409,634) (Affx-52342012). There are 795 of the heterozygous genotype C/CA of rs527905870 among White British individuals.
